# Negative Associations between Corpus Callosum Midsagittal Area and IQ in a Representative Sample of Healthy Children and Adolescents

**DOI:** 10.1371/journal.pone.0019698

**Published:** 2011-05-19

**Authors:** Hooman Ganjavi, John D. Lewis, Pierre Bellec, Penny A. MacDonald, Deborah P. Waber, Alan C. Evans, Sherif Karama, The Brain Development Cooperative Group

**Affiliations:** 1 Department of Psychiatry, McGill University, Montreal, Quebec, Canada; 2 McConnell Brain Imaging Centre, Montreal Neurological Institute, McGill University, Montreal, Quebec, Canada; 3 Department of Neurology and Neurosurgery, McGill University, Montreal, Quebec, Canada; 4 Department of Psychiatry, Harvard Medical School, Children's Hospital, Boston, Massachusetts, United States of America; 5 Department of Psychiatry, Douglas Mental Health University Institute, McGill University, Montreal, Quebec, Canada; Chiba University Center for Forensic Mental Health, Japan

## Abstract

Documented associations between corpus callosum size and cognitive ability have heretofore been inconsistent potentially owing to differences in sample characteristics, differing methodologies in measuring CC size, or the use of absolute versus relative measures. We investigated the relationship between CC size and intelligence quotient (IQ) in the NIH MRI Study of Normal Brain Development sample, a large cohort of healthy children and adolescents (aged six to 18, n = 198) recruited to be representative of the US population. CC midsagittal area was measured using an automated system that partitioned the CC into 25 subregions. IQ was measured using the Wechsler Abbreviated Scale of Intelligence (WASI). After correcting for total brain volume and age, a significant negative correlation was found between total CC midsagittal area and IQ (*r* = −0.147; *p* = 0.040). Post hoc analyses revealed a significant negative correlation in children (age<12) (*r* = −0.279; *p* = 0.004) but not in adolescents (age≥12) (*r* = −0.005; *p* = 0.962). Partitioning the subjects by gender revealed a negative correlation in males (*r* = −0.231; *p* = 0.034) but not in females (*r* = 0.083; *p* = 0.389). Results suggest that the association between CC and intelligence is mostly driven by male children. In children, a significant gender difference was observed for FSIQ and PIQ, and in males, a significant age-group difference was observed for FSIQ and PIQ. These findings suggest that the correlation between CC midsagittal area and IQ may be related to age and gender.

## Introduction

The corpus callosum (CC) is the largest fiber tract in the human brain and is responsible for much of the communication between the two hemispheres. It is a topographically organized structure which connects homologous areas in the two cerebral hemispheres [1]. It has been found to be important in the transfer of visual, motoric, somatosensory, and auditory information [2].

Compared to other mammals, the human brain is highly lateralized. This allows for a greater degree of specialization of the hemispheres, and potentially to the intellectual capacity of humans. According to Banich and Brown [3], it is more efficient for information to be processed by a single hemisphere, provided the task is simple enough. For more complex tasks, use of both hemispheres provides parallel processing power, and may be preferable. Thus, reliance on the CC for interhemispheric communication is a function of the task and possibly the capacity of the individual hemisphere to compute the information. Not surprisingly, it has been postulated that the size and/or morphology of the CC could be related to individual cognitive ability. Both the theories and the associated data are inconsistent, however. Consistent with the theory that a larger CC confers greater processing power, Luders et al. [4] found IQ to be positively correlated with the thickness of the posterior CC in healthy adults. In contrast, in a study of 138 individuals aged six to 88, Peterson et al. [5] found the anterior portion of the CC to be negatively associated with cognitive ability, although overall CC area was not. Allin et al. [6] found a negative correlation between the area of the posterior CC and IQ in a sample of healthy teens, a finding later replicated by Hutchinson et al. [7]. These findings were interpreted to mean that a smaller CC represents a more “high performance” brain that relies less on interhemispheric communication [6], [7]. Still other studies finding no relationship between CC size and IQ in typically developing individuals [5], [8], [9], [10].

In order to clarify the relationship between CC size and IQ in typically developing individuals, we investigated this relationship in a relatively large representative sample of healthy children and adolescents compiled by the NIH MRI Study of Normal Brain Development described by Evans et al. [11]. The NIH Study used a mixed cross-sectional and longitudinal design to create a database with MRI, clinical, and behavioral data from children aged 4.6 to 18.3 years. In order to improve generalizability, a population-based epidemiologic sampling strategy was used to obtain a sample reflecting the population of the 2000 United States Census. Data were collected at three time points (Visits 1, 2, and 3, spaced two years apart). Only the cross-sectional data from Visit 1 (release 4.0) were used for analysis in this study.

## Methods

### Ethics statement

Written informed consent was obtained from all participants or their parents when appropriate. The institutional review board of McGill University (Data Coordinating Center) approved this study. Institutional review board approval was also obtained from the six Pediatric Study Centers: Children's Hospital, Boston; Children's Hospital Medical Center of Cincinnati; University of Texas Health Science Center at Houston/University of Houston - Texas Medical Center Annex; Washington University, St. Louis; Children's Hospital of Philadelphia; University of California, Los Angeles.

### Sampling and recruitment

A representative sample of 431 healthy subjects was recruited into the study at the following study centers: Children's Hospital Boston, Cincinnati Children's Hospital Medical Center, University of Texas Houston Medical School, UCLA Neuropsychiatric Institute and Hospital, Children's Hospital of Philadelphia, and Washington University. At each site, recruitment was targeted to be in line with the US Census data and monitored regularly to ensure a representative sample according to age, gender, ethnicity, and socioeconomic status. As this was a study of normal brain development, exclusion criteria included history of an Axis I psychiatric disorder (with some exceptions), abnormal neurological examination, medical illness with CNS implications, history of head trauma with loss of consciousness >30 minutes or abnormal imaging, history of systemic malignancy requiring chemotherapy or CNS radiotherapy, an IQ<70, known intrauterine exposure to substances thought to alter brain structure and/or function, complicated labor and/or delivery, and family history of inherited neurological disorders or medical disorders with known CNS implications. Further details about exclusion criteria can be found in Evans, et al. [11] and Waber et al. [12]. Data from all sites were transferred electronically to the Data Coordinating Center at the Montreal Neurological Institute.

Mean age of puberty is estimated to range from 12.2 to 12.8 years for females in the US, with breast development and menarche occurring somewhat earlier [13]. For males, pubertal onset occurs with testicular enlargement which occurs at a mean age of 12 [14]. In light of this, we defined children as being below age 12 and adolescents age 12 and above.

### IQ measurement

Cognitive ability was measured using the full four-subtest version of the Wechsler Abbreviated Scale of Intelligence (WASI) [15]. This version of the WASI provides for estimation of Verbal IQ (VIQ), Performance IQ (PIQ), and Full-Scale IQ (FSIQ). VIQ score is composed of the Vocabulary and Similarities subtests; PIQ is composed of the Matrix Reasoning and Block Design subtests. All testing was performed on the day of or within a few days of image acquisition. Children below age six were not included in the analysis because they did not have WASI scores (WASI norms start at 6).

### MRI acquisition and CC measurement

MRI images were acquired as per the NIH MRI Study of Normal Brain Development protocol described by Evans et al. [11]. Briefly, a 3D T1-weighted (T1W) Spoiled Gradient Recalled (SPGR) echo sequence was obtained with 1 mm isotropic data acquired sagittally from the entire head. Slice thickness of approximately 1.5 mm were obtained from GE scanners due to their limit of 124 slices. T2-weighted (T2W) and proton density weighted (PDW) images were acquired using a 2D multi-slice (2 mm) dual echo fast spin echo (FSE) sequence. Subjects who were unable to tolerate this procedure received a fallback protocol that consisted of shorter 2D acquisitions with slice thicknesses of 3 mm. The same (or an equivalent protocol for GE scanners – see comment above) was used at each site. While summarized below, details are outlined in Table 3 of Evans et al. [11]. Importantly, two forms of data were collected at each site and used to calibrate the various scanners and make sure their outputs were comparable:

- The American College of Radiology (ACR) phantom: this phantom contains various compartments which provide information on intensity non-uniformity over a flat intensity field and geometric distortion over a grid pattern (collected approximately monthly).
- The living phantom: one normal adult volunteer was scanned at all sites using the full MRI acquisition protocol. This database of real brain MRIs provided information on inter-site variability in brain-related measures such as tissue contrast in raw MRI signal, tissue relaxation properties and derived morphological measurements (collected annually) [11].

Raw MRI images were processed through the CIVET pipeline, developed at the MNI for automated structural image analysis [16], [17], [18].

Of the 431 subjects recruited for the NIH study, 392 had MRI acquisitions that were of acceptable quality. Of these 392 subjects, 33 were under six years of age and had no IQ score (norming for the WASI begins at age 6). Of the remaining 359 subjects, 107 had T1W fallback protocols, and these were excluded from the study because they did not provide adequate data for these analyses. Scans for the remaining 252 subjects underwent a blinded visual quality control (QC) procedure independently performed by two investigators (HG and SK). This visual inspection identified an additional 54 cases with inadequate image quality, leaving a final sample size of 198 subjects for this analysis.

**Figure 1:**
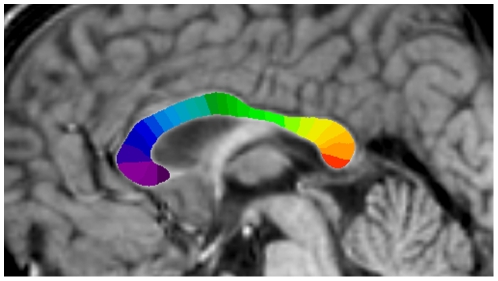
The midsagittal corpus callosum divided into 25 segments.

The boundary and 25 divisions of the CC were identified on the midsagittal slice of the Talairach-like MNI152 template, an average of 152 T1-weighted images [19]; the MNI152 template was then registered to the T1-weighted image of each subject to measure the corresponding CC areas. The boundary and divisions of the CC on the MNI152 template were established using a semi-automated procedure based on Clarke's method [20]. The procedure was as follows. The midsagittal slice of the template was extracted and upsampled to 0.1 mm×0.1 mm. An intensity-based flood-fill was used to establish an initial boundary of the CC. An implementation of the active contour algorithm was then used to transform this initial estimate into a smoothed boundary at the centre of the gradient at the edge of the CC. Lines were then radiated from the centroid of the CC at 1-degree intervals, and for each line that crossed the CC, the point midway between the two points of intersection with the boundary was determined, and the shortest length line that crossed the CC through that point was determined. The curved line that passed through the midpoint of each of these lines and extended to the CC boundary on either end defined the midline. The midline was divided into 25 equal length segments. The shortest length lines that crossed the CC at the points defined by these midline segments defined the subregion boundaries. The subregions are identified as 1 to 25 from rostral to caudal as illustrated in Figure 1. The procedure is described in detail in Lewis et al. [21].

CC subregion measurements for a subject were obtained by warping the MNI152 template to the subject's *T*_1_ volume, and using the resultant transform to overlay the template CC on the subject's *T*_1_ volume. First, the rotation and translation components of the linear transform determined by CIVET were used to align the subject *T*_1_ to the MNI152 template. The scaling component of the CIVET linear transform was then composed with the CIVET non-linear transform, and the left-right deformations eliminated in the area of the CC, to provide an initial transform for a refined nonlinear registration process. To eliminate distortions due to differences in lateral structure, a 7 mm wide portion of the brain mask, centered on the midsagittal slice, was used limit the evaluation of the fit. To reduce distortions caused by variability in the fornix, the fornix was blurred on the template. To constrain left-right deformations in the area of the CC, minctracc was provided with a feature volume pair in which voxels within the CC in slice *i* had the value 1000*i, and voxels elsewhere were zero. For the MNI152 template this was achieved by concatenating binarized versions of the template CC. For the subject, an equivalent volume was constructed by dilating the MNI152 feature volume, warping it onto the subject with the initial transform derived from CIVET, and using the mean intensity within the resultant volume to threshold out everything but the CC. Using this feature volume pair, the nonlinear registration process extended the CIVET nonlinear registration from a step-size of 4 to a step-size of 1, utilizing the gradient in the T1-weighted volumes to achieve a good fit of the CC boundary. The transform resulting from the nonlinear registration process was then applied to the high-resolution MRI152 CC volume, and the area of each of the subdivisions of the CC on the subject was measured.

### Correlation analyses – total corpus callosum midsagittal area

**Figure 2:**
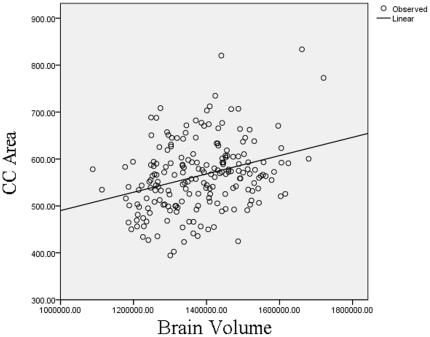
Regression of total corpus callosum area against total brain volume.

Computation of basic descriptive statistics was performed using SPSS [22]. Partial correlation coefficients (two-tailed) corrected for total brain volume and age were calculated between cognitive ability and total CC area. Correcting for total brain volume was done to control for the potential confounding effect of total brain volume on the association between CC area and IQ as there is a known association between total brain volume and IQ [23], [24] as well as between total brain volume and CC area (see Figure 2).

### Correlation analyses – corpus callosum segments

Analysis of individual CC segments was performed using MATLAB [25]. The correlations between the measures of IQ and the CC midsagittal surface area at each segment were calculated after controlling for linear effects of age, gender, and total brain volume. Group differences between the correlation coefficients were also assessed for the following subgroup pairs: males vs females, children vs adolescents, male children vs female children, male adolescents vs female adolescents, male children vs male adolescents, and female children vs female adolescents. The significance of the partial correlation coefficients as well as of the difference between correlation coefficients was assessed using permutation tests. The permutations were applied on residual CC measures on the reduced model, i.e. after least-square fitting of the factors of no interest [26]. A number (B = 10,000) of independent permutation samples were generated to derive an estimate of the false positive rate (*p*-value) under the null hypothesis of independence between the measures of IQ and the CC area, as described by Bellec et al. [27]. To correct for multiple comparisons, the permutation samples were fed into an estimation procedure of the false-discovery rate (FDR) associated with each observed *p*-value [28]. Because the same permutations of the residuals were used for all CC segments, this procedure accounted for the potential spatial dependencies between tests.

### General linear model - IQ interactions with age and gender

IQ interactions with age and gender were investigated using a general linear model with age, gender, and total brain volume as covariates. When males and females were analyzed separately, gender was not used in the model. An *age-group by IQ* interaction term was added to the model to determine if there was a difference between children in adolescents in the relationship between CC segment area and IQ. To do a formal statistical analysis of the effect of age on the CC segment area-IQ relationship, age as a continuous variable was used instead of age-group. A *gender by IQ* interaction term was used to test the effect of gender on the CC segment area-IQ relationship. To determine if the *age by IQ* interaction varies by gender, an *age by IQ by gender* interaction was used in the model. Correction for multiple comparisons was performed using the false-discovery rate method as described above.

## Results

### Sample characteristics

Table 1 shows the demographic and neuropsychological characteristics of the analyzed sample of 198 subjects and the original sample. Age was the only demographic characteristic found to be different in the two groups. This is due to elimination of children under six years of age. No significant differences between the samples were found for proportion of males, socioeconomic status, ethnicity, and IQ scores.

**Table 1:** Demographic and FSIQ characteristics of original NIH sample and analyzed sample.

Characteristic	Original NIH Sample	Analyzed Sample	Statistics
Sample size	n = 431	n = 198	
Age (years)	10.4±3.8	11.8±3.5	*t* = 4.54, *p*<0.001
Proportion males	48%	43.4%	*χ^2^* = 1.15, *p* = 0.28
Proportion with low/medium/high adjusted SES*	22.9%/41.6%/35.5%	23.2%/37.9%/38.9%	*χ^2^* = 1.31, *p* = 0.52
Proportion of Whites/African Americans/Other**	78.9%/9.2%/11.9%	74.2%/8.6%/17.2%	*χ^2^* = 5.25, *p* = 0.07
FSIQ***	110.7±12.5	111.1±12.1	*t* = 0.41, *p* = 0.68

When appropriate, means ± standard deviations are provided.

*p*-values were calculated using the Student's t-test or chi-square test where appropriate.

*Based on the US Department of Housing and Urban Development method for comparing family income levels as a function of regional costs of living.

**The ‘Other’ category includes American Indian, Alaskan Native, Asian, Native Hawaiian or Other Pacific Islander, and those for which ethnicity or race was not provided or for which parents came from different racial or ethnic background.

***WASI IQ data available for only 380 subjects out of 431 that were initially recruited.

The balance of subjects from each study site and their demographic characteristics are shown in Table 2. There were no main effects of scanner site on age, FSIQ, or total CC area.

**Table 2:** Balance of subjects and demographic characteristics from each study site.

	Site 1	Site 2	Site 3	Site 4	Site 5	Site 6	Statistics
**Subjects**	46	37	44	28	35	8	
**Gender**	Male = 20	Male = 17	Male = 20	Male = 15	Male = 12	Male = 2	*χ^2^* = 3.64
	Female = 26	Female = 20	Female = 24	Female = 13	Female = 23	Female = 6	*p* = 0.60
**Mean age**	11.1±3.5	12.0±3.7	11.9±3.2	12.0±3.9	12.5±3.3	11.0±3.7	*F*_5,191_ = .726
							*p* = 0.61
**Mean FSIQ**	111.2±14.9	114.9±10.9	109.8±10.2	108.6±11.3	111.7±10.5	113.0±16.8	*F*_5,191_ = 1.303
							*p* = 0.26
**CC Total**	545.75±61.4	560.62±76.2	561.4±68.1	575.5±78.3	593.8±87.4	530.6±60.1	*F*_5,191_ = 1.99
							*p* = 0.08

### Relationship among age, gender and corpus callosum midsagittal area

Corpus callosum area was positively correlated with both total brain volume (*r* = 0.308; *p*<0.001) and age (*r* = 0.390; *p*<0.001). To determine if age is associated with CC area independent of brain size, partial correlations were calculated correcting for total brain volume. The positive correlation between age and CC area was actually enhanced by this correction (*r* = 0.424; *p*<0.001). Consistent with previous findings [29], we found that males have a larger absolute CC area (*p* = 0.028) but females have a higher CC area to total brain volume ratio (*p* = 0.005).

### Relationship between scanner site and either IQ or corpus callosum midsagittal area

No main effect of site was shown for full scale IQ (*p* = 0.246), VIQ (*p* = 0.177), PIQ (*p* = 0.361), or CC area (*p* = 0.256).

### Correlation between IQ and total corpus callosum area

Partial correlation coefficients (adjusted for total brain volume and age) were calculated based on the entire sample of 198 subjects. Subsequent analyses were performed with the sample partitioned into two groups based on gender (86 males and 112 females) and age (106 children and 92 adolescents). Table 3 shows the partial correlations between total CC area and FSIQ, PIQ, and VIQ in the combined sample, and divided samples based on gender and age.

**Table 3:** Correlations between total corpus callosum midsagittal area and FSIQ, VIQ, and PIQ correcting for total brain volume and age.

	All Ages (n = 198)			Age<12 (n = 106)			Age≥12 (n = 92)		
M and F	FSIQ	VIQ	PIQ	FSIQ	VIQ	PIQ	FSIQ	VIQ	PIQ
(n = 198)									
CC area	−0.147*	−0.104	−0.119	−0.279**	−0.163	−0.280**	−0.005	−0.031	0.055
Males	FSIQ	VIQ	PIQ	FSIQ	VIQ	PIQ	FSIQ	VIQ	PIQ
(n = 86)									
CC area	−0.231*	−0.178	−0.198	−0.498**	−0.312*	−0.520**	0.096	−0.022	0.226
Females	FSIQ	VIQ	PIQ	FSIQ	VIQ	PIQ	FSIQ	VIQ	PIQ
(n = 112)									
CC area	−0.083	−0.053	−0.056	−0.062	−0.032	−0.043	−0.097	−0.059	−0.074

*p≤0.05.

**p≤0.01.

As Table 3 shows, all the correlations were negative, indicating that smaller total CC area was associated with higher scores. For the total sample, the correlation between total CC area and FSIQ was weak but statistically significant. The correlation was stronger, however, for children analyzed alone, but correlations were not statistically significant for the adolescents. When VIQ and PIQ subscales were considered separately, the correlation was significant only for PIQ.

The magnitude of the associations also differed by gender. There were no significant correlations were found between any of the IQ measures in either children or adolescents for females considered alone. Among the males, there was a negative correlation between total CC area and FSIQ which was attributable to those who were 12 or under, but not the adolescents. Again, this association was primarily attributable to the correlation between total CC area and PIQ.

To verify that the negative correlation between CC area and IQ in male children was due to a CC area effect and not simply larger relative cerebral hemispheres, the correlation was calculated removing total brain volume as correcting variable. Larger brain size is associated with higher IQ in the subgroup of male children (*r* = 0.307; *p* = 0.043), but the negative correlation between CC area and IQ remains significant when correcting for age but not total brain volume (*r* = −0.399; *p* = 0.007).

### Correlation between IQ and corpus callosum segments

**Figure 3:**
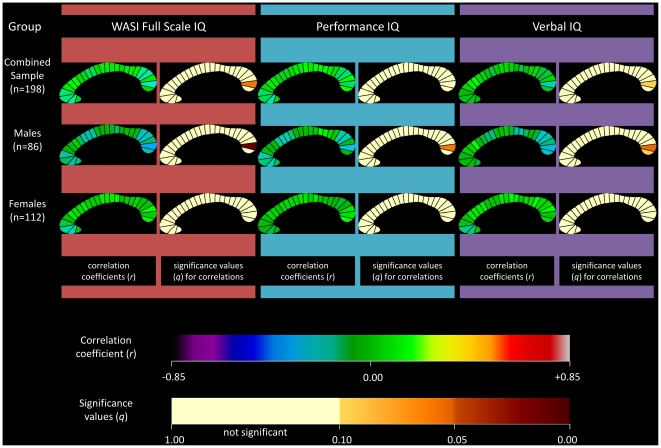
Correlations between corpus callosum midsagittal area and IQ: combined sample and sample partitioned by gender. Partial correlation coefficients and associated significance values for Full-Scale IQ measurements are shown in the first and second columns of callosal maps. Correlation coefficients and associated significant values for Performance IQ are shown in the third and fourth columns, and for Verbal IQ in the fifth and sixth columns. The combined sample is shown in the first row, the males in the second row, and the females in the third row. The upper color bar encodes the *r*-values that depict the magnitude and direction of correlations, and the lower color bar encodes the level of significance.

**Figure 4:**
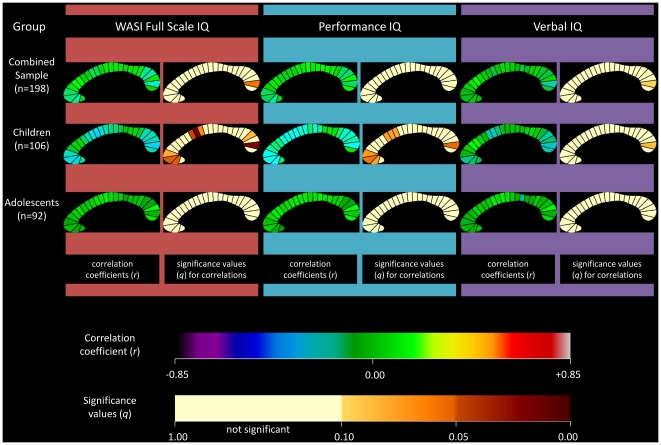
Correlations between corpus callosum midsagittal area and IQ: combined sample and sample partitioned by age. Partial correlation coefficients and associated significance values for Full-Scale IQ measurements are shown in the first and second columns of callosal maps. Correlation coefficients and associated significant values for Performance IQ are shown in the third and fourth columns, and for Verbal IQ in the fifth and sixth columns. The combined sample is shown in the first row, the children in the second row, and the adolescents in the third row. The upper color bar encodes the *r*-values that depict the magnitude and direction of correlations, and the lower color bar encodes the level of significance.

Partial correlations were also calculated for each of the twenty-five CC segments individually. The correlation coefficients and associated significance values for FSIQ, PIQ, and VIQ are displayed as callosal maps in Figure 3. A trend is defined as meeting an FDR threshold of 10% and significance is defined as meeting a threshold of 5%. The maps indicate that the negative correlation between CC area and FSIQ in the combined sample was mostly driven by the posterior region, specifically segment 24 which approached significance (*r* = −0.196; *q* = 0.06; *p* = 0.007). When partitioned by gender, this negative correlation was observed in the males group (*r* = −0.348; *q* = 0.011; *p* = 0.001). When partitioned by age, children demonstrated a negative correlation at several sites (Figure 4). Segments 10, 11, and 24 were significant at 5% FDR, and 2, 3, 4, 12, and 22 showed a trend towards significance. The adolescent group did not show any significant correlations.

**Figure 5:**
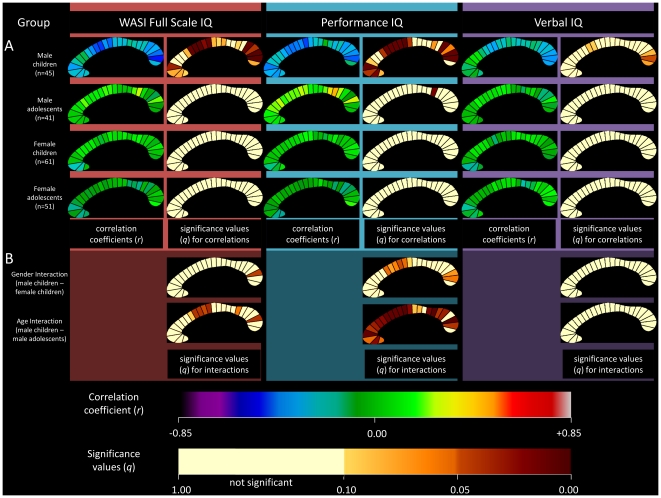
Correlations between corpus callosum midsagittal area and IQ: combined sample and sample partitioned by age and gender. A. Partial correlation coefficients and associated significance values for Full-Scale IQ measurements are shown in the first and second columns of callosal maps. Correlation coefficients and associated significant values for Performance IQ are shown in the third and fourth columns, and for Verbal IQ in the fifth and sixth columns. The male children are shown in the first row, the males adolescents in the second row, the female children in the third row, and the female adolescents in the fourth row. B. Gender and age interactions. Gender interaction in children is shown in the first row and age-group interaction is shown in the second row. The upper color bar encodes the *r*-values that depict the magnitude and direction of correlations, and the lower color bar encodes the level of significance for the correlation coefficients and the interactions.

The most significant negative correlations were found for the subgroup of male children (Figure 5). Several segments demonstrated a negative correlation, with the most significant segments corresponding to the truncus and the genu (posterior). The highest correlation (*r* = −0.498; *q* = 0.005; *p* = 0.0007) was observed between segment 24 and PIQ. Female children did not show any correlations in any segment, nor did female or male adolescents.

**Figure 6:**
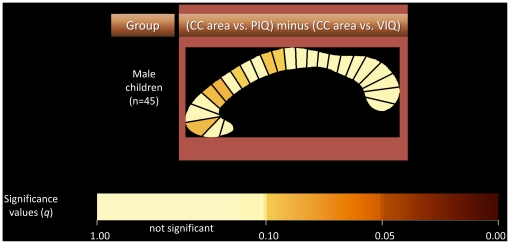
Direct comparison of VIQ and PIQ correlations in male children.

A non-parametric direct correlation comparison procedure (with permutations) between the PIQ and VIQ correlations demonstrated a trend (false discovery rate = 0.1) towards PIQ being significantly more negatively associated with CC area than VIQ in several segments (Figure 6). Similar findings were produced when looking at the association between PIQ and CC segments after controlling for VIQ.

### IQ interactions with gender

*Gender by IQ*, *age-group by IQ*, and *age by IQ* interactions were estimated to determine the effect of gender, age-group, and age on the relationship between CC segment area and IQ. No overall *gender by IQ* interaction could be demonstrated. However, in children, a *gender by IQ* interaction was shown for a region in the posterior CC. This interaction appeared to be driven by PIQ (Figure 5).

### IQ interactions with age-group and age as a continuous variable

For the group as a whole, no significant *age-group by IQ* interaction was demonstrated. However, in males, an *age-group by IQ* interaction was shown at several segments. This interaction appeared to be driven by PIQ (Figure 5).

**Figure 7:**
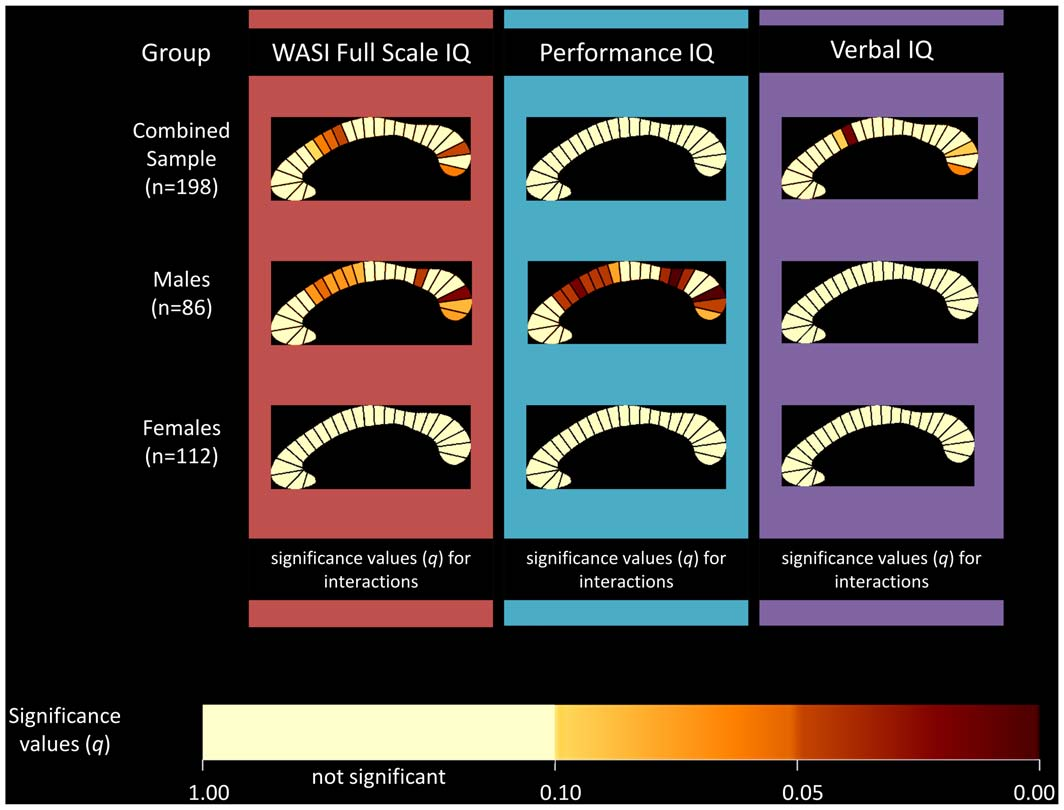
Age by IQ interaction: combined sample and sample partitioned by gender. A general linear model was used with an *age by IQ* interaction term to examine the interaction between IQ and age as a continuous variable. Significance values are displayed as callosal maps with Full-Scale IQ results shown in the first column, Performance IQ results in the second column, and Verbal IQ results in the third column. Values for the combined sample are shown in the first row and results for males and females are shown in the second and third rows respectively. The color bar encodes the level of significance for the interactions.

When age was examined as a continuous variable, a significant *age by IQ* interaction was seen at several segments in the group as a whole (Figure 7). When males and females were analyzed separately, the *age by IQ* interaction was only seen in males. Again, the interaction appeared to be largely driven by PIQ (Figure 7).

When the *age by IQ by gender* interaction term was added to the full model, several segments demonstrated significance, statistically verifying that the *age by IQ* interaction varies by gender. This was the case for FSIQ, PIQ, and VIQ.

### Impact of not controlling for brain volume in young males

For the vast majority of CC segments, not controlling for brain volume in young males left intact the negative direction of the association between CC segment area and all three measures of cognitive ability. However, not controlling for brain volume slightly decreased the level of significance of the associations (yet keeping above threshold the significance of many segments for FSIQ and PIQ). No segment areas were found to have a significant positive association with measure of cognitive ability.

## Discussion

This study demonstrates that there is a negative correlation between CC area and cognitive ability. Further, it shows that this relationship is primarily driven by males and varies with age. These results may shed light on some of the reasons why the relationship between CC area and cognitive ability is not clear. One possibility is the methodological differences in the way CC area is measured. For instance, while CC area was measured in this and other studies [5], [6], [9], [10], Luders et al. [4] used CC thickness instead. In some cases, the CC was subdivided into four segments [6], [9]; other studies used five segments [5], [7]. Other investigators used voxel-based morphometry to examine the relationship between white matter in the CC and cognitive ability.

Secondly, in contrast to the sample used in this study, the vast majority of studies assessing relationships between CC size and cognitive ability were based on samples of convenience. Differences in age, gender, socioeconomic status, and other demographic variables could influence the findings. However, the most likely explanation for contrasting results in the literature is that the relationship between CC area and cognitive ability is a dynamic one that changes over time and is influenced by a number of developmental factors. The age range in our study was six to 18, and the negative correlation was only found in the younger six to 12 group. It is worth noting that the two studies that demonstrated a negative correlation between CC area and cognitive ability were performed with relatively young individuals, aged 15 and 19 [6], and 14 to 25 [7]. The mean age in the Luders [4] study which showed a positive correlation was 28.1 for males and 28.8 for females. Peterson's study [5] which showed no overall correlation used subjects from age six to 88 (mean 25.5±22.2). Haier's study [8] which also did not show a correlation included subjects aged 18 to 37 (mean 27±5.9). The children in our study who showed a negative correlation between CC area and IQ were therefore younger than the subjects used in other studies. The age range of the adolescents in our study who did not show any correlation roughly correspond in age to the subjects used in the studies of Allin et al. [6] and Hutchison et al. [7] which showed a negative correlation between CC area and IQ. Therefore, while age variability does not fully explain the different findings, it does seem to be an important factor with the studies finding a negative correlation between CC area and cognitive ability tending to include younger subjects.

In the current study, in males, an *age-group by IQ* interaction was found at several segments throughout the length of the CC. The relative absence of an association in the adolescent group could indicate that in healthy individuals it is the *rate* of CC growth that varies with cognitive ability as opposed to final CC area in the mature adult brain. If the negative correlation between CC area and cognitive ability is due to the relative dependence on a single hemisphere and a reduced need for interhemispheric connectivity, the smaller CC areas could represent a greater proportion of smaller diameter, slower-conducting transcallosal fibers [30]. If increased cognitive ability is related to increased interhemispheric communication, then a smaller CC could represent an increased rate of pruning, which presumably would result in more efficient communication by reducing unnecessary crosstalk. Animal models have shown that pruning of callosal axons is a normal part of postnatal development [31], [32], and interruption of this process by depriving somatosensory input or other manipulations leads to altered neural activity [33], [34], [35], [36], [37], [38].

As Hutchinson et al. point out [7], another possible explanation for age-related differences in the association between CC area and cognitive ability is the nature of the cognitive demands in older age. They suggest that as one ages, cognitive demands become increasingly complex and more likely to require interhemispheric processing. This could be a reason why a positive correlation was found between CC thickness and cognitive ability in the initial work of Luders et al. [4]. To verify this, a longitudinal study would have to be performed.

In addition to age being an important factor in the negative association between CC area and cognitive ability, we find a contrast in the effect of gender. No association between CC area and cognitive ability in either children or adolescents was found in females. There are well-known gender differences in cognitive ability. Males perform better at certain visuospatial tasks including mental rotation and tracking objects through space, while females are stronger in some verbal tasks including synonym generation and verbal fluency. Pre-pubertal females are superior at in mathematics, but this relationship reverses to the advantage of males post-puberty and into old age [39], [40]. It has recently been reported that males and females rely on different pathways that lead to the same level of cognitive ability. In a study of 48 male and female subjects of similar IQ, Haier et al. [41] qualitatively showed that compared to men, women show more white matter and fewer grey matter areas associated with cognitive ability. In males, IQ to grey matter correlations are strongest in the frontal and parietal lobes while in females, the strongest correlations are in the frontal lobe including Broca's area. In children, we demonstrated that there is a *gender by IQ* interaction in some CC segments. To our knowledge, this study is the first to show a gender difference in the association between CC area and cognitive ability.

With respect to the specific segments of the CC that are most highly associated with cognitive ability, our study appears relatively consistent with the findings of Hutchison et al. [7] and Allin et el. [6], despite methodological differences in CC area measurement and segmentation. Our findings demonstrate that a small region in the splenium of the CC is the region most consistently negatively associated with cognitive ability. Hutchinson et al. [7] and Allin et al. [6] demonstrated a negative association between the posterior CC and IQ. Our data also show three separate clusters of segments along the CC that are negatively correlated with cognitive ability. In male children, the posterior region (approximately segments 19 to 25) shows a strong negative correlation with FSIQ and PIQ. The region roughly links homologous occipital, parietal, and temporal cortical areas. For VIQ, the negative correlation in the posterior region is restricted to segments 23 to 25. The second region that also shows a negative correlation with cognitive ability is approximately within segments eight to 14. This region of the CC roughly links homologous premotor cortical regions. Again, PIQ is contributing most to the negative correlation (Figure 5). Finally, segments 1 to 5, corresponding to the prefrontal cortex also shows a negative correlation with cognitive ability. The negative correlation in that region is seen with FSIQ and PIQ, but not with VIQ.

It is noteworthy that directly contrasting the PIQ and VIQ correlations did not reveal significant differences between them (differences only reached the level of trend). Whether this is due to lack of power to detect a difference or to the fact that no genuine significant difference exists between PIQ and VIQ correlations with the areas of CC segments cannot be established at this point. Whatever may be the case, it is well known that PIQ and VIQ are highly correlated [42]. In light of all this, the apparent difference observed here between findings for PIQ and VIQ should be viewed with a certain degree of healthy skepticism. Having said this, negative correlations between areas of CC segment and FSIQ being largely mediated by PIQ would be consistent with the work of Hutchinson et al. [7]. Assuming as genuine the differences between PIQ and VIQ in terms of their association with CC area, the underpinnings of a differential PIQ relationship to interhemispheric connectivity remains unclear. It is possible that the cognitive processing required of the Matrix Reasoning and Block Design tests require a greater integration of functions dependent upon left and right hemisphere regions than verbal tasks that contribute to VIQ. Language function tends to be left-lateralized, and there is some evidence that lateralization is greater in males [43], [44]. If young males with large CC areas have less efficient interhemispheric communication, then it is conceivable that these individuals would not perform as well on the PIQ subtests, whereas VIQ performance would be relatively unaffected. This, of course, is purely speculative and would have to be examined in future studies. However, the recent finding that a larger CC is associated with increased language lateralization is consistent with that notion [45]. In a study conducted in parallel using the same NIH database (preliminary data from our study was first presented in abstract form in 2009 [46]), Luders et al. [47], examined the association between CC thickness and intelligence. They report mainly positive associations in females, and negative associations confined to the splenium in males. The only associations that survived after correcting for multiple comparisons, however, were the negative associations in the overall sample. Our study similarly demonstrates a negative association between CC area and cognitive ability in the overall sample which survives correction for multiple comparisons. It also demonstrates a robust negative association in males that survives correction for multiple comparisons that is present in various regions throughout the CC. Our findings also differ in that no positive association was found in females.

While speculative, there are a few possible reasons why these two studies offered similar yet somewhat diverging results. First, Luders and colleagues measured thickness while we measured cross-sectional area. Although the same structure was measured in the two studies, the measurement of CC thickness and CC area could provide slightly different information. It is noteworthy that cross-sectional area has been shown to strongly correlate with the number of fibers passing through the callosum [48]. Second, according to the description of Luders' method, CC thickness was measured at 100 points equally spaced along the midline of the callosum within each subject. In contrast, we defined the divisions on the template and then fit the template to each individual subject. This helped maximize the chances that measurements were made from the same regions of the callosum in all individuals as this method is rather insensitive to local differences in CC shape, such as an elongated rostrum, which may shift measurement points between subjects. Third, in contrast to Luders' study, we corrected for total brain volume. In other words, what we estimated was the association between CC area and IQ while keeping brain size constant. Not correcting for total brain volume could be viewed as providing complementary information. We opted to control for potential confounding effects of total brain volume for reasons stated in the methods section regarding known associations between brain volume and IQ as well as between brain volume and CC area.

In summary, this study examines the relationship between CC area and cognitive ability in a relatively large representative sample of children and adolescents from the US population. Thus, it does not suffer from the potential selection biases that characterize samples of convenience or self-selected samples. We have shown fairly strong correlations that remain significant after correcting for multiple comparisons, and we have demonstrated the effect of age and gender on these associations. To our knowledge, this study represents the most robust evidence available to date demonstrating the negative association between CC area and cognitive ability in healthy children and adolescents. In interpreting the data, however, one needs to remember that we have demonstrated an association, and proposed potential mechanisms, however, these need to be verified in future studies.

The data in this study suggest that the association between CC area and cognitive ability is a function of age and gender. We propose that age, developmental factors, and gender influence the dynamic relationship between callosal morphology and cognitive ability, which may help account for the existence of conflicting data in the literature. Our analyses suggest that there is little to no correlation between CC area and cognitive ability in post-pubertal individuals, or in females. However, we demonstrate that in the young male brain, a negative correlation exists. We put forward two possible explanations: (1) in the developing male brain, decreased CC area is associated with increased cognitive ability by way of accelerated pruning resulting in more efficient interhemispheric communication, or (2) the decreased presence of large diameter, faster conducting axons signifies increased hemispheric capacity and decreased need for recruitment of the opposite hemisphere. We propose that the female brain does not depend on either of these mechanisms. Our findings point to the need to consider age and gender as moderators in future studies looking at CC area and cognitive ability.
